# Environmental enrichment induces behavioural disturbances in neuropeptide Y knockout mice

**DOI:** 10.1038/srep28182

**Published:** 2016-06-16

**Authors:** Florian Reichmann, Vanessa Wegerer, Piyush Jain, Raphaela Mayerhofer, Ahmed M. Hassan, Esther E. Fröhlich, Elisabeth Bock, Elisabeth Pritz, Herbert Herzog, Peter Holzer, Gerd Leitinger

**Affiliations:** 1 Research Unit of Translational Neurogastroenterology, Institute of Experimental and Clinical Pharmacology, Medical University of Graz, Universitätsplatz 4, 8010 Graz, Austria; 2 Research Unit Electron Microscopic Techniques, Institute of Cell Biology, Histology and Embryology, Medical University of Graz, Harrachgasse 21, 8010 Graz, Austria; 3 Neuroscience Division, Garvan Institute of Medical Research, 384 Victoria Street, Darlinghurst, Sydney, NSW 2010, Australia

## Abstract

Environmental enrichment (EE) refers to the provision of a complex and stimulating housing condition which improves well-being, behaviour and brain function of laboratory animals. The mechanisms behind these beneficial effects of EE are only partially understood. In the current report, we describe a link between EE and neuropeptide Y (NPY), based on findings from NPY knockout (KO) mice exposed to EE. Relative to EE-housed wildtype (WT) animals, NPY KO mice displayed altered behaviour as well as molecular and morphological changes in amygdala and hippocampus. Exposure of WT mice to EE reduced anxiety and decreased central glucocorticoid receptor expression, effects which were absent in NPY KO mice. In addition, NPY deletion altered the preference of EE items, and EE-housed NPY KO mice responded to stress with exaggerated hyperthermia, displayed impaired spatial memory, had higher hippocampal brain-derived neurotrophic factor mRNA levels and altered hippocampal synaptic plasticity, effects which were not seen in WT mice. Accordingly, these findings suggest that NPY contributes to the anxiolytic effect of EE and that NPY deletion reverses the beneficial effects of EE into a negative experience. The NPY system could thus be a target for “enviromimetics”, therapeutics which reproduce the beneficial effects of enhanced environmental stimulation.

Environmental enrichment (EE) refers to a housing condition which provides animals with enhanced physical, social and/or cognitive stimulation opportunities^1^. EE has been shown to induce a wide range of beneficial behavioural alterations such as anxiolysis and stress resilience^2^,^3^,^4^. Furthermore, EE ameliorates various disease phenotypes including Alzheimer’s disease^5^, Parkinson’s disease^6^, Huntington’s disease^7^, traumatic brain injury^8^ and epilepsy^9^. The key question is how EE, a manipulation that is neither pharmacological nor genetic, but purely environmental, induces such strong biological effects. Understanding the molecular mechanisms underlying the behavioural effects of EE may foster the development of novel therapeutic interventions, based on the idea that molecules mediating the beneficial effects of EE are potential drug targets. Ideally, compounds acting on these targets will mimic the benefits of environmental stimulation and thus ameliorate disease symptomatology^7^,^10^,^11^.

Neuropeptide Y (NPY), a 36-amino acid peptide, is one of the most abundant neuropeptides in the brain. The peptide has been implicated in many physiological processes such as food intake, energy homeostasis and circadian rhythm, but also in fundamental brain functions related to cognition and emotion^12^. Specifically, both central NPY administration^13^,^14^,^15^ and hippocampal as well as amygdalar NPY overexpression reduce anxiety^16^,^17^,^18^ in rodents. Recently, NPY has caught attention because of its ability to promote stress resilience and its therapeutic potential for post-traumatic stress disorder (PTSD)^12^,^19^,^20^,^21^. For example, intra-amygdalar administration of NPY prevents the deleterious behavioural consequences of stress^22^, NPY protects from behavioural disruption in animal models of PTSD^23^,^24^,^25^,^26^, and cerebrospinal fluid concentrations of NPY are reduced in combat veterans suffering from PTSD^27^,^28^. NPY is widely distributed in the mammalian central nervous system^29^ including amygdala and hippocampus, two brain areas which play a central role in the modulation of emotional-affective behaviour and stress processing^30^. Although NPY and EE induce very similar effects, a role for NPY in the beneficial effects of EE has not yet been examined. In the present study, we therefore used genetically modified mice lacking NPY to analyse the relevance of this neuropeptide to the behavioural, molecular and morphological consequences of EE.

## Results

### EE-housed wildtype (WT), but not NPY knockout (KO) mice, are less anxious in the elevated plus maze test (EPM)

**Figure 1:**
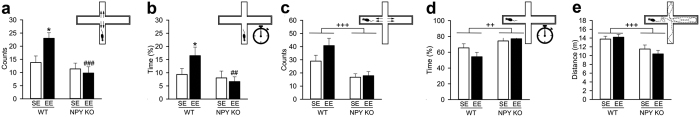
NPY KO abolishes the anxiolytic effects of EE in the EPM. (**a**) The number of open arm entries (interaction: F_(1,32)_ = 5.150, P = 0.030; post-hoc test, *P < 0.05 vs. WT/SE; ^###^P < 0.001 vs. WT/EE) and (**b**) the time spent on the open arms of the EPM show an anxiolytic effect of EE only in WT animals (interaction: F_(1,32)_ = 3.015, P = 0.092; post-hoc test, ^##^P < 0.01 vs. WT/EE). (**c**) The number of closed arm entries (significant main effect of genotype: F_(1,32)_ = 18.283, P < 0.001), (**d**) the time spent on the closed arms (significant main effect of genotype: F_(1,32)_ = 9.286, P = 0.005) and (**e**) total distance travelled (significant main effect of genotype: F_(1,32)_ = 15.140, P < 0.001) reveal an hypo-explorative and hypo-locomotive phenotype of NPY KO mice. The time spent on the open and closed arms is expressed as percentage of the 5-min test duration. Values represent means ± SEM. ^++^P < 0.01, ^+++^P < 0.001 for main effect: NPY KO vs. WT independently of housing condition; n = 9–10.

To analyse the relevance of NPY for the behavioural effects of EE, we first performed the EPM and found a strong anxiolytic effect of EE in WT animals (Fig. 1a,b) in terms of an increased number of entries into the open arms (23.0 ± 2.1 vs. 13.8 ± 2.5) and an increased time spent on the open arms (16.5 ± 3.2% vs. 9.4 ± 2.3%). In contrast, these effects were completely absent in NPY KO mice. Other readouts of the EPM were not altered by the housing conditions but influenced by the NPY genotype. Specifically, NPY KO decreased closed arm entries (17.4 ± 2.0 vs. 34.2 ± 3.7) and total distance travelled (10.9 ± 0.6 m vs. 14.0 ± 0.5 m), while time spent on the closed arms was increased (75.8 ± 3.3% vs. 60.5 ± 4.0%), suggesting a hypo-explorative and hypo-locomotive phenotype of the KO mice (Fig. 1c–e).

### NPY KO mice are more anxious in the open field test (OF) than WT mice

**Figure 2:**
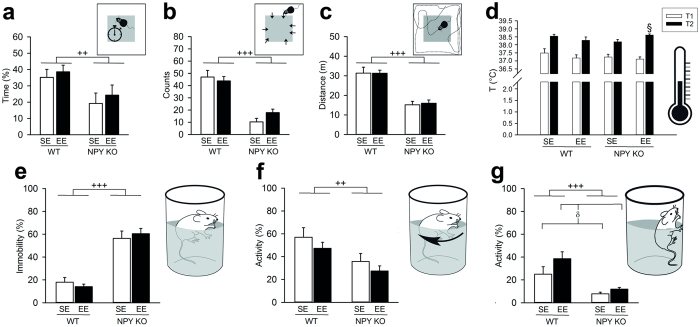
Effects of EE and NPY KO in the OF test, stress-induced hyperthermia test and FST. Analysis of (**a**) the time spent in the central zone of the OF (significant main effect of genotype: F_(1,35)_ = 7.712, P = 0.009) and (**b**) the number of entries into the central zone (significant main effect of genotype: F_(1,35)_ = 64.696, P < 0.001) reveals an anxiogenic phenotype of NPY KO mice. (**c**) Reduced traveling distance indicates a hypo-locomotive phenotype of NPY KO mice (significant main effect of genotype: F_(1,35)_ = 54.025, P < 0.001). (**d**) In the stress-induced hyperthermia test EE-housed NPY KO mice have higher stress-induced temperatures (T2; interaction: F_(1,36)_ = 5.595, P = 0.024; post-hoc test, ^§^P < 0.05 vs. NPY KO/SE) while basal rectal temperatures (T1) are similar between treatment groups. (**e**–**g**) In the FST a higher percentage of time spent immobile (significant main effect of genotype: F_(1,35)_ = 83.788; P < 0.001) as well as reduced times spent swimming (significant main effect of genotype: F_(1,35)_ = 9.842; P = 0.003) and climbing (significant main effect of genotype: F_(1,35)_ = 18.856; P < 0.001) indicate a depression-like phenotype of NPY KOs. In contrast, EE increases climbing behaviour (significant main effect of housing condition: F_(1,35)_ = 7.086; P = 0.012), but does not alter swimming or immobility. The time spent in the central zone of the OF (**a**) is expressed as percentage of the 5-min test duration. The measured times in E, F and G are expressed as percentage of the 6-min test duration. Values represent means ± SEM. ^++^P < 0.01, ^+++^P < 0.001 for main effect: NPY KO vs. WT independently of housing condition; ^δ^P < 0.05 for main effect: EE vs. SE independently of genotype; n = 9–10.

To gain further insight into the behavioural actions of EE and NPY KO, the OF was performed. In this test the NPY KO mice displayed an anxious phenotype which corroborates the behavioural features seen in the EPM: NPY KO decreased the time spent in the central area (21.6 ± 4.4% vs. 36.9 ± 3.1%) and the number of central area entries (13.9 ± 2.2 vs. 45.4 ± 3.2), suggesting elevated anxiety levels (Fig. 2a,b). Furthermore, like in the EPM, total traveling distance (Fig. 2c) was reduced in NPY KO mice (15.5 ± 1.2 m vs. 31.3 ± 1.7 m), confirming the hypo-locomotive phenotype.

### EE-housed NPY KO mice show an increased thermal stress response

Measurement of the basal temperature in mice with a rectal probe represents a stressor that causes an increase in body temperature by 1–1.5 °C within 15 min^31^. We found that this stress-induced hyperthermia (T2) was increased in EE-housed NPY KO mice (38.6 ± 0.1 °C vs. 38.2 ± 0.1 °C), but not in EE-housed WT mice, relative to the stress-induced hyperthermia in the respective standard environment (SE)-housed mice (Fig. 2d). The basal rectal temperature (T1) did not differ between the different housing conditions and genotypes, confirming the elevated autonomic stress response of the EE-housed mutant mice.

### NPY KO mice display depression-like behaviour

To analyse another component of emotional-affective behaviour, the forced swim test (FST) was performed. Time spent immobile in the FST reflects behavioural despair and is commonly used as readout of depression-like behaviour. The active behaviours during the test can be divided into swimming and climbing behaviour, which, according to pharmacological and lesion studies, predominantly are under control of different (serotonergic vs. catecholaminergic) neurotransmitter systems^32^. We observed that NPY KO renders the mice more immobile (58.3 ± 4.0% vs. 16.0 ± 2.3%) indicating a depression-like phenotype (Fig. 2e). As a consequence both the time spent swimming (31.9 ± 4.3% vs. 52.2 ± 4.9%) as well as the time spent climbing (9.8 ± 1.1% vs. 31.8 ± 4.6%) were shortened in the NPY KO mice (Fig. 2f,g). EE enhanced climbing time (25.9 ± 4.5% vs. 16.4 ± 3.8%) in a genotype-independent manner, suggesting altered catecholaminergic signalling.

### EE-housed NPY KO mice have an impaired short-term memory

**Figure 3:**
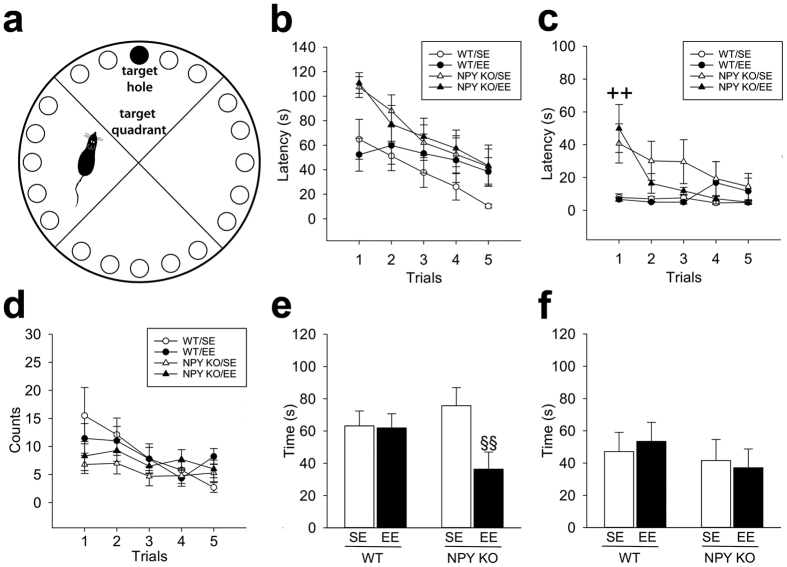
EE impairs short-term memory of NPY KO mice. (**a**) Schematic illustration of the Barnes maze. (**b**) The latency to find the target hole decreases for all mice upon repeated trials (significant main effect of trial: F_(2.575,90.133)_ = 15.621, P < 0.001). NPY KO mice have higher target latencies (significant main effect of genotype: F_(1,35)_ = 6.651, P = 0.014). (**c**) The latency to explore the first hole is higher for NPY KO mice during the first training trial (interaction: F_(1.798,62.931)_ = 9.322, P < 0.001; Post-hoc test, ^++^P < 0.01 for main effect: NPY KO vs. WT independently of housing condition). (**d**) The number of primary errors tends to be lower in NPY KO mice (main effect of genotype: F_(1,35)_ = 3.134, P = 0.085). (**e**,**f**) EE-housed NPY KO mice spend less time in the target quadrant during the probe trial (interaction: F_(1,34)_ = 3.552, P = 0.068; post-hoc test, ^§§^P < 0.01 vs. NPY/SE mice) 48 h (**e**), but not 9d (**f**) after the last training trial. Values represent means ± SEM; n = 9–10.

EE is known to improve cognitive performance of rodents in the Morris water maze^33^. We therefore examined the effect and interaction of NPY and EE on learning and memory readouts in a related spatial memory task, the Barnes Maze test (BM) (Fig. 3a). During the training phase mice of all groups became faster in finding the location of the target hole upon repeated trials (Fig. 3b). However, the NPY KO animals displayed, on average, longer target hole latencies than WT mice (70.8 ± 4.8 s vs. 43.8 ± 4.3 s), which usually indicates a learning deficit but may here rather reflect the hypo-locomotive, hypo-explorative and anxiogenic phenotype of NPY KO mice seen in the EPM and OF. Supporting this idea, the latency to explore the first hole after starting the test session differed considerably between WT and NPY KO mice, the latter exhibiting freezing behaviour and very slow movements during the first training trials (Fig. 3c; Supplementary Movie S1). The view that NPY KO mice did not suffer from a learning deficit is also supported by the number of primary errors made (Fig. 3d), which tended to be lower in the KO animals (6.6 ± 0.6 vs. 8.7 ± 0.9).

In the first probe trial (removal of the box 48 h after the last training session), the time spent in the target quadrant, a parameter not affected by hypolocomotion or anxiety, was significantly shortened in EE-housed NPY KO mice (36.4 ± 10.6 s vs. 75.8 ± 11.2 s), indicating a memory deficit (Fig. 3e, Supplementary Movie S2). During the second probe trial (7 days after probe trial 1), however, all mice had a poor performance (time in target zone only slightly above chance level), without any differences between housing conditions and genotypes (Fig. 3f).

### EE-housed NPY KO mice prefer other EE items than WT mice

**Figure 4:**
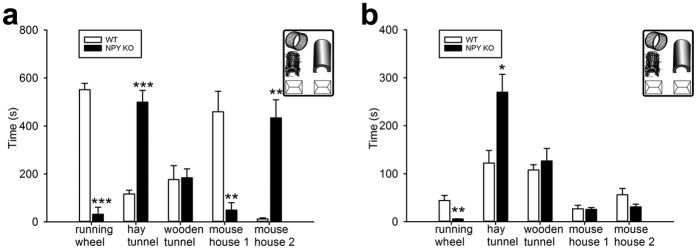
NPY KO mice prefer other EE items than WT mice. (**a**) During the dark phase of the light/dark cycle, NPY KO mice spend on average less time on the running wheel (t = 13.040, d.f. = 10), but more time with the hay tunnel (t = −7.411, d.f. = 6.012) than WT mice. While the wooden tunnel is used in a similar manner by both groups, NPY KO and WT mice show a differential preference for the two plastic mouse houses in the cage (mouse house 1: t = 4.523, d.f. = 6.297; mouse house 2: t = −5.596, d.f. = 5.038) (**b**) When NPY KO mice are individually placed in a novel EE cage, they also avoid the running wheel (t = 3.683, d.f. = 8) and use the hay tunnel for a longer time period (t = −3.237, d.f. = 8) than WT mice. The other cage objects were used for the same amount of time. Data in A are derived from six time frames within a 24 h observation period of one EE cage/genotype. Values represent means ± SEM; *P < 0.05, **P < 0.01, ***P < 0.001 vs. WT; n = 5–6.

To determine whether WT and NPY KO mice use the EE objects to the same extent, we measured the time each EE object is used over a 24 h period (12 h light/12 h dark). During the light phase, WT and NPY KO mice were asleep for most of the time and, with the exception of occasional food and water intake, they showed only little interest to interact with the cage objects. In contrast, during the dark phase, mice from both cages became active and started to interact. Strikingly, WT mice used the running wheel almost continuously during the dark phase, while NPY KO mice, although also awake, used it only for a limited amount of time (551.0 ± 26.7 s vs. 31.8 ± 29.5 s; Fig. 4a). NPY KO mice appeared to prefer the hay tunnel, because they spent significantly more time with this object than the WT mice (499.1 ± 49.1 s vs. 116.8 ± 15.7 s). The wooden tunnel and the plastic mouse houses were in sum used for the same amount of time by both lines, although WT mice showed a preference for mouse house 1 (459.2 ± 85.2 s vs. 49.1 ± 31.0 s) and NPY KO mice for mouse house 2 (434.1 ± 75.2 s vs. 12.3 ± 4.6 s; Fig. 4a).

When WT or NPY KO mice were individually placed in a novel EE cage for 10 min (Fig. 4b), the differential EE item preference was similar to the preference observed during the 24 h analysis. Specifically, WT mice spent more time interacting with the running wheel (44.3 ± 10.5 s vs. 5.5 ± 0.5 s), while NPY KO mice spent more time interacting with the hay tunnel (269.9 ± 37.3 s vs. 122.3 ± 26.3 s). The wooden tunnel and both plastic mouse houses were used for the same amount of time (Fig. 4b).

### EE increases *Npy* mRNA expression in the limbic system

**Figure 5:**
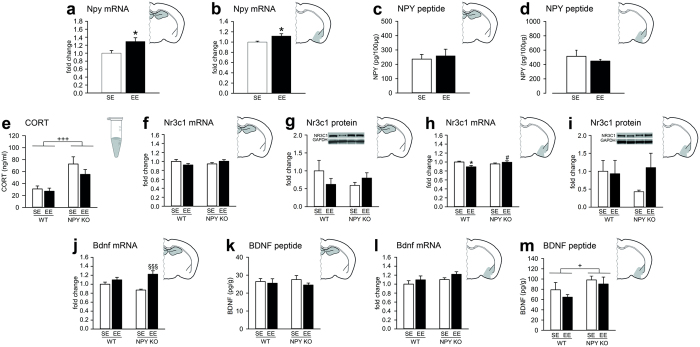
Effects of EE on NPY expression, CORT levels, NR3C1 and BDNF expression. (**a**,**b**) EE increases hippocampal (t = −2.572, d.f. = 10; *P < 0.05) and amygdalar (t = −2.456, d.f. = 6.422) Npy mRNA levels. (**c**,**d**) Hippocampal and amygdalar NPY peptide levels are not influenced by housing condition. (**e**) NPY KO mice have increased circulating CORT levels (significant main effect of genotype: F_(1,35)_ = 17.869, P < 0.001). (**f**) EE tends to reduce hippocampal Nr3c1 mRNA levels (interaction: F_(1,20)_ = 3.729, P = 0.068; post-hoc test: n.s.) and (**g**) hippocampal NR3C1 protein levels in WT, but not in NPY KO mice. (**h**) Similarly, WT/EE mice have lower amygdalar Nr3c1 mRNA levels (F_(1,20)_ = 7.153, P = 0.015; post-hoc test, ^#^P < 0.05 vs. WT/EE). (**i**) Amygdalar NR3C1 protein levels are not affected by genotype or housing condition. (**j**) After EE, mice from both genotypes show elevated hippocampal Bdnf mRNA levels (significant main effect of housing condition: F_(1,20)_ = 16.924, P = 0.001), but the increase is higher in NPY KO mice (interaction: F_(1,20)_ = 5.493, P = 0.030; post-hoc test, ^§§§^P < 0.001 vs. NPY KO/EE). (**k**) Hippocampal BDNF peptide levels and (**l**) amygdalar Bdnf mRNA levels are not altered by the experimental conditions. (**m**) Amygdalar BDNF protein levels are higher in NPY KO mice (significant main effect of genotype: F_(1,19)_ = 5.367, P = 0.032). The inserts in panels (**g**,**i**) depict representative Western blots. The mRNA and protein level values are expressed as fold changes normalized to WT/SE. Peptide levels were normalized to the total protein content of the tissue homogenates. Values represent means ± SEM; ^+^P < 0.05, ^+++^P < 0.001 for main effect: NPY KO vs. WT independently of housing condition; n = 6 for panels (**a**–**d**,**f**,**h**,**j**–**m**); n = 9–10 for panel E; n = 4 for panel (**g**,**i**).

To test for an involvement of NPY in the anxiolytic effects of EE at the molecular level, we measured *Npy* mRNA expression and peptide levels in limbic brain areas of WT mice. In both the hippocampus and the amygdala, EE increased *Npy* mRNA expression (Fig. 5a,b), but did not change the corresponding peptide levels (Fig. 5c,d), suggesting a higher NPY turnover rate. As expected, tissue from NPY KO mice did not show any NPY mRNA expression (data not shown).

### EE reduces amygdalar glucocorticoid receptor (*Nr3c1*) expression in WT, but not NPY KO mice

It has been suggested that the anxiolytic effect of EE relates to the plasma concentration of the stress hormone corticosterone (CORT) and the central expression of its receptor NR3C1^34^, which tempted us to analyse the role of these molecules in the observed behavioural phenotypes. As seen in Fig. 5e, basal circulating CORT levels were increased in NPY KO mice independently of housing conditions, indicating an overactive hypothalamic-pituitary-adrenal axis or an impaired hypothalamic feedback in the mutants. In contrast, NR3C1 expression in the amygdala-hippocampus network depended on housing and genotype, with a very similar pattern of effects at both mRNA and protein level. In WT animals, NR3C1 mRNA and peptide levels were reduced after EE, whereas EE had no effect on NR3C1 levels in NPY KO animals (Fig. 5f–i). Statistics showed a significant difference between WT/SE and WT/EE animals in amygdalar mRNA levels (Fig. 5h), whereas no statistical significant difference was seen in hippocampal *Nr3c1* expression (Fig. 5f), hippocampal protein levels (Fig. 5g) and amygdalar NR3C1 protein levels (Fig. 5i). These changes in receptor expression may imply altered EE-induced stress hormone effects on the amygdala-hippocampus network of WT, but not NPY KO mice.

### EE and NPY KO alter brain-derived neurotrophic factor (BDNF) levels in a region-dependent manner

BDNF is another factor, which is thought to contribute to the behavioural effects of EE^35^. We therefore evaluated the region-specific BDNF expression under the current experimental conditions. We found that hippocampal *Bdnf* mRNA levels were elevated after environmental stimulation, but only in NPY KO mice (Fig. 5j), while hippocampal BDNF peptide levels (Fig. 5k) were not modified by the experimental variables, suggesting an EE-induced increase in BDNF turnover. In contrast, in the amygdala, EE did not change *Bdnf* mRNA expression (Fig. 5l), but amygdalar BDNF peptide levels were higher in the mutant mice suggesting aberrant amygdalar synaptic plasticity (Fig. 5m).

### EE and NPY KO have diverse effects on the synaptic ultrastructure of the dentate gyrus

**Figure 6:**
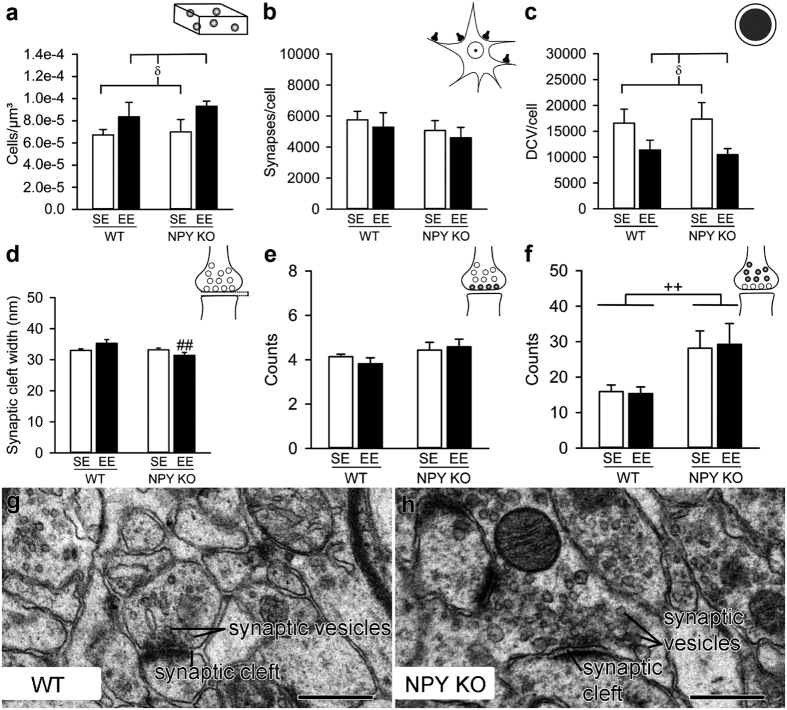
Effects of EE and NPY KO on ultrastructural features of the DGpl. (**a**) EE increases the numerical density of neurons (significant main effect of housing condition: F_(1,15)_ = 5.028, P = 0.040), but (**b**) does not change the number of synapses per neuron. (**c**) In contrast, the number of DCVs per neuron is significantly lower after EE (significant main effect of housing condition: F_(1,15)_ = 6.135, P = 0.026). (**d**) EE increases synaptic cleft width in WT mice, but decreases the width in NPY KO mice (interaction: F_(1,15)_ = 5.933, P = 0.028; post-hoc test, ^##^P < 0.01 vs. WT/EE). (**e**) Housing condition and genotype do not alter the number of docked synaptic vesicles, but (**f**) the number of undocked synaptic vesicles is higher in NPY KO mice (significant main effect of genotype: F_(1,15)_ = 9.003, P = 0.009). (**g**,**h**) Representative electron micrographs of synapses in the DGpl of an EE-housed WT animal (**g**) and an EE-housed NPY KO animal (**h**). Scale bars 400 nm. Values represent means ± SEM. ^++^P < 0.01 for main effect: NPY KO vs. WT independently of housing condition; ^δ^P < 0.05 for main effect: EE vs. SE independently of genotype. Data in (**a**–**c**) are derived from image pairs using 20 counting frames of 5.5 × 5.5 μm^2^ from 4–5 animals/group; n = 10 for panels (**d**–**f**).

Finally, we analysed the interaction of NPY and EE on an ultrastructural level. For this, the polymorph cell layer of the dentate gyros (DGpl) was analysed because EE alters hippocampal synaptic plasticity^36^ and the DGpl represents the major source of hippocampal NPY^37^. As expected, enhanced environmental stimulation resulted in a number of effects. EE increased the numerical density of neurons (Fig. 6a), indicating spatial remodelling of the DG. While the number of synapses per neuron was not changed by EE (Fig. 6b), the number of dense-core vesicles (DCV) per neuron was significantly reduced (Fig. 6c), suggesting increased neuropeptide release. Interestingly, EE increased synaptic cleft width in WT mice, but decreased it in NPY KO mice (Fig. 6d) indicating that NPY signalling determines the way EE affects this parameter. The number of docked small synaptic vesicles (Fig. 6e) was left unchanged by EE and NPY KO, whereas the number of undocked small synaptic vesicles was increased in NPY KO animals, suggesting an increase in the reserve pool of synaptic vesicles (Fig. 6f–h). We also measured presynaptic membrane length and postsynaptic density length, which remained stable across the experimental groups (Supplementary Fig. S1).

## Discussion

Our results provide evidence that behavioural, molecular and morphological effects of EE are at least partly linked to NPY. We observed that global NPY gene KO prevents the anxiolytic effect of EE and the concomitant change of *Nr3c1* expression in the amygdala. Moreover, we found that EE has adverse consequences for NPY mutant mice as demonstrated by elevated stress-induced hyperthermia and impaired spatial memory, changes that are paralleled by enhanced hippocampal *Bdnf* expression and synaptic plasticity in the DGpl as demonstrated with electron microscopy.

A role of NPY in the neurobiological manifestations of EE has not yet been systematically investigated although both NPY and EE have similar beneficial effects on rodent behaviour^3^,^15^. Here, we provide evidence for a role of NPY in EE-induced anxiolysis. In agreement with previous research^2^, our EE paradigm had an anxiolytic effect in WT mice as shown in the EPM. This EE-induced anxiolysis was absent in NPY mutant mice. Moreover, housing conditions affected the NPY system as *Npy* gene expression in both hippocampus and amygdala of WT mice was increased by EE. Altered *Npy* gene expression after EE was also shown previously^38^,^39^, suggesting that NPY modulates some of the neurobiological effects of EE in these brain regions. Since NPY peptide levels in the amygdala-hippocampus network remained unaltered after EE (this study), we surmise that the neuronal release of NPY was increased, which in turn stimulated *Npy* gene transcription and NPY turnover. This conclusion is supported by the finding that the number of DCVs, which contain neuropeptides^40^, is reduced after EE.

Interestingly, EE failed to modify anxiety levels in the OF test. Although at first glance unexpected, it needs to be kept in mind that anxiety phenotyping with the most common behavioural tests such as EPM, OF or light/dark box test measures different aspects of anxiety^41^. Accordingly, our results indicate that the EE paradigm used affects some but not all dimensions of anxiety.

The anxiolytic phenotype of EE-housed WT animals was accompanied by altered glucocorticoid signalling. Specifically, we found lowered *Nr3c1* expression in the amygdala-hippocampus network in conjunction with unchanged CORT levels, relative to SE-housed WT mice. The emerging change in the ligand-receptor ratio may dampen the influence of stress hormones on the limbic system. The result is anxiolysis because the downregulation of limbic NR3C1 mitigates the anxiogenic action of CORT^42^. Notably, the observed decreased *Nr3c1* expression may be directly linked to increased NPY signalling, given that a recent study has shown that intranasal NPY administration reduces stress-induced hippocampal NR3C1 expression^43^.

Apart from modulating the anxiolytic effects of EE, NPY gene knockout turned EE into a negative experience. This conclusion is based on adverse behavioural traits of EE-housed NPY KO mice, namely, increased stress-induced hyperthermia, impaired spatial memory and avoidance of some EE items. We hypothesize that three mechanisms may be of relevance to these negative effects. The first mechanism, in our opinion, relates to a particular interaction between BDNF and NPY. We and others^44^ found hippocampal *Bdnf* expression to be elevated by EE in WT mice, but in the present study discovered that *Bdnf* mRNA levels were considerably higher in EE-housed NPY KO mice. While enhanced *Bdnf* mRNA levels after EE are considered a key factor in EE-induced beneficial behavioural effects^45^, in the current work an exaggerated hippocampal *Bdnf* transcription in EE-housed mutants was associated with some behavioural deterioration. Although unexpected, this finding needs to be seen in the light of a disturbed interrelationship between NPY and BDNF. *In vitro* BDNF enhances the NPY content of cultured neurons, and neuronal cultures from BDNF KO mice do not show NPY upregulation^46^. Conversely, peripheral NPY administration has been demonstrated to alter brain neurotrophin expression in rats, and NPY increases BDNF mRNA and peptide levels in primary rat cortical neurons^47^. In NPY KO mice these BDNF/NPY interactions cannot take place, and thus we speculate that some of the effects of BDNF to facilitate hippocampal neuronal plasticity depend on NPY. Exaggerated *Bdnf* expression in EE-housed NPY KO mice may reflect an attempt to compensate for the missing downstream mediator NPY.

The second mechanism that may underlie the negative impact of EE in NPY KO mice is a change in the synaptic architecture of the DGpl. In line with a previous study from our group^48^, EE increased synaptic cleft width in the DGpl of WT mice, the major source of hippocampal NPY^49^. In EE-housed NPY KO mice, however, synaptic cleft width was altered in the opposite direction, i.e. it was reduced, pointing to increased synaptic gain in the DGpl. The relationship between reduced synaptic cleft width and impairment of spatial memory appears counterintuitive, given the common association of increased synaptic cleft width and negative behavioural phenotypes^50^,^51^. However, it needs to be kept in mind that it depends on the brain area investigated, its network structure and the functions of the neurons involved whether increased synaptic transmission efficiency translates into beneficial or adverse behavioural effects. The DGpl synapses under investigation include those between the dendrites of glutamatergic mossy cells or GABAergic interneurons (HIPP and HICAP cells) and collaterals from the mossy fibre tract (=axons of granule cells) and/or axons from extrahippocampal regions including the medial septal nucleus, the diagonal band nucleus of Broca, the supramammillary area, the locus coeruleus, ventral tegmental area and the raphe nucleus^52^. Reduced synaptic cleft width in the DGpl could signify, for instance, an enhanced influence of the afferent fibres on hilar cells and their functions. It is believed that a key function of the HIPP cells, the GABAergic interneuron subset expressing *Npy*^49^, and also of mossy cells is the regulation of granule cell activity^53^. The granule cells are a key component of the memory pathway, and thus disturbance of neurons regulating their activity will unavoidably interfere with memory formation.

A third potential mechanism, which may contribute to the negative effects of EE in NPY KO mice, is the differential use of the EE items provided. Unlike WT mice, NPY KO mice hardly ever use the running wheel but extensively interact with the hay tunnel, which may be related to their anxiety-prone phenotype. It has been suggested that physical exercise on a running wheel is an important component of EE, able to induce neurobiological and beneficial behavioural effects on its own^54^,^55^. If the running wheel is not used, as was the case for the NPY KO mice in this study, the mice cannot profit from the potential benefits of exercise. The strong avoidance of the running wheel by the knockout mice fits with the observed hypo-locomotive phenotype in the behavioural tests and may indicate that NPY influences the motivation to do physical exercise. This behavioural trait is a potentially confounding factor in the analysis of NPY’s contribution to the neurobiological effects of EE.

In confirmation of a previous report^56^ we found that NPY KO mice displayed a distinct behavioural phenotype independently of housing condition. Specifically, NPY KO rendered the mice anxious, hypo-explorative and hypo-locomotive. Furthermore, the mutant mice displayed enhanced depression-like behaviour in the FST, which is supported by another report^57^. In contrast NPY KO had no genuine effect on learning and memory in the BM independently of housing condition. In line with this finding, NPY KO mice neither showed learning and memory impairments in the passive avoidance and hole-board tasks nor gross locomotor abnormalities in the accelerated rotarod test^56^. The changes in emotional-affective behaviour seen in mutant mice were associated with heightened basal plasma CORT levels as well as enhanced amygdalar BDNF concentrations. Given the anxiogenic properties of CORT^42^, dysregulation of the hypothalamic-pituitary-adrenal axis in the NPY KO animals may contribute to the anxiogenic phenotype. Similarly, enhanced amygdalar BDNF levels may cause anxiety, based on the findings that anxiety and amygdalar spinogenesis are enhanced in mice overexpressing BDNF^58^. We also found that NPY mutants had an increased number of reserve pool vesicles independently of housing condition, suggesting a previously unidentified influence of NPY on small vesicle cycling in the DGpl.

Some effects of EE were not influenced by the NPY genotype. At the behavioural level we found EE to increase the climbing behaviour in the FST, which may be related to a change in catecholaminergic neurotransmitter signalling in both genotypes^32^. At the morphological level we observed that, in the DGpl, the numerical density of cells was increased after EE, while the number of DCVs/cell was decreased. EE is known to increase adult neurogenesis in the dentate gyrus^53^, but this process is confined to the granular cell layer (DGgl). The newly formed progenitor cells migrate exclusively into the DGgl, extending their dendrites towards the molecular layer. Thus, the increased cell density in the DGpl might be a result of DGpl compression by increased neurogenesis in the DGgl, but not an effect related to cell proliferation in the DGpl itself. As previously described^48^, the number of DCVs in WT mice was lower after EE, but here we found that this effect is also present in the DGpl of NPY KO mice. We thus speculate that EE stimulates DCV turnover in both genotypes, but this process may be without a functional consequence in NPY KO mice, because of the absence of NPY.

The current study was carried out with germ-line NPY KO mice which due to their constitutive null mutation may have neurodevelopmental abnormalities and/or compensatory changes due to the developmental as well as adult absence of NPY. Although beyond the scope of this work, our hypothesis that NPY is crucial for the behavioural effects of EE would be strengthened by experiments incorporating pharmacological and/or genetic manipulation of the adult NPY system. For example, it would be worthwhile to determine whether EE-housed WT mice chronically treated with NPY antagonists fail to benefit from EE or, conversely, whether SE-housed WT mice treated with NPY or NPY agonists show EE-like changes. Other potential experiments include the analysis of SE- or EE-housed mice with brain-region-specific NPY overexpression or deletion as well as rescue experiments such as chronic NPY administration in the NPY KO mice.

In summary, the current data are consistent with the hypothesis that the resilience-inducing properties of EE are linked to NPY. Accordingly, NPY deletion abolished the anxiolytic effect of EE which in WT mice was accompanied by enhanced NPY signalling in the amygdala-hippocampus network. In addition, NPY knockout turned enhanced environmental stimulation into a negative experience. Although developmental compensations in germ-line NPY gene KO mice may be a confounding factor, our data indicate that, if such adaptations occurred, they were insufficient to balance the deficit in NPY. From a translational perspective, our findings support the view that gene x environment interactions may play an important role for individuals with polymorphisms of the *Npy* gene^59^,^60^. Furthermore, the study suggests that the NPY system could be a target for “enviromimetics”^11^, therapeutics which mimic or enhance the beneficial effects of enhanced environmental stimulation.

## Materials and Methods

### Experimental animals

The study was carried out with male WT (n = 45) and NPY KO (n = 45) mice on a mixed C57BL/6:129/SvJ (1:1) background. Germ line NPY −/− mice in which the entire coding sequence including the initiation start codon was removed were generated as reported^56^. The presence or deletion of NPY was verified by PCR^56^. These homozygous NPY KO and their corresponding WT mice were obtained from the Neurobiology Research Program of the Garvan Institute of Medical Research (Sydney, Australia) and bred at the Institute of Experimental and Clinical Pharmacology of the Medical University of Graz (Graz, Austria). Animals were housed under controlled temperature (set point 22 °C), relative air humidity (set point 50%) and light conditions (lights on at 0600 h, lights off at 1800 h). Tap water and standard laboratory chow were provided ad libitum throughout the study.

### Ethical statement

The experimental procedures and number of animals used were approved by an ethical committee at the Federal Ministry of Science, Research and Economy of the Republic of Austria (BMWF-80.104/2-BrGT/2007 and BMWF-66.010/0037-II/3b/2013) and conducted according to the Directive of the European Parliament and of the Council of 22 September 2010 (2010/63/EU). The experiments were designed and performed in such a way that the number of animals used was minimized.

### Study design

For the experiments, 6–10-week-old WT or NPY KO mice were housed in groups of five either in SE or EE for 10–11 weeks as described previously^61^. Briefly, SE-housed mice were kept in small cages measuring 36.5 × 20.7 × 14.0 cm, while EE-housed mice were kept in large cages measuring 59.0 × 38.0 × 20.0 cm. In addition, EE-housed mice had access to various enrichment items including a running wheel, different kinds of tunnels, plastic or cardboard mouse houses and nesting material, which were all absent in the SE cages^61^. At the end of the differential housing period we either evaluated emotional-affective behaviour (experiment 1) or learning and memory (experiment 2).

For experiment 1, WT and NPY KO mice were submitted to a behavioural battery during week 11 of the differential housing period to assess anxiety (EPM, day 1), locomotion and exploration (OF, day 2), stress-induced hyperthermia (day 3) and depression-like behaviour (FST, day 4). Twenty-four hours afterwards mice were euthanized with an overdose of pentobarbital (150 mg/kg IP) and blood and brain tissues were collected for molecular analysis.

Experiment 2: A separate cohort of mice was habituated (week 9, day 1) trained (week 9, day 2 + 3) and tested (2 probe trials; week 9 day 5 and week 10 day 5) on the BM. Thirty min after the second probe trial mice were euthanized and brains were processed for electron microscopy.

A separate cohort of mice (n = 5/group) was used to evaluate the interaction of EE-housed mice with their cage objects. For this, WT and NPY KO mice were habituated to the EE cage for 10 days. On day 10, the behaviour of the mice in their cage was video recorded for 24 h (12 h light/12 h dark under red light). Six 10-min time frames within the 24 h period (start point: 10 min, 1 h, 2 h, 4 h, 6 h and 8 h after the lights went off) were selected to analyse the average time each object was used by at least one mouse. According to our EE paradigm/week 2, the cage objects used were a running wheel (Dehner, Graz, Austria), a wooden tunnel (Dehner), a hay tunnel (Dehner) and two transparent red plastic mouse houses (Ehret, Tulln, Austria)^61^. On day 11, mice were individually placed in a novel EE cage and their interaction time with the same cage objects was measured for 10 min.

### EPM

The EPM was used to assess anxiety-like behaviour. For this, mice were placed on the central platform of the maze and their behaviour was videotaped and tracked with VideoMot 2 software (TSE Systems, Bad Homburg, Germany) for 5 min. Number of entries into and time spent on the open and closed arms as well as total distance travelled was quantified to evaluate anxiety, exploration and locomotion.

### OF

For the OF, mice were individually placed in the centre of a grey plastic box, and their behaviour was videotaped and tracked for 5 min. Number of entries into and time spent in the central area were used as anxiety readouts, while total distance travelled was measured to analyse locomotion.

### Stress-induced hyperthermia

This test consisted of a series of 2 rectal temperature measurements using a digital thermometer (BAT-12, Physitemp Instruments, Clifton, New Jersey, USA) equipped with a rectal probe for mice representing a physical stressor. Fifteen min after the first measurement (T1), a second measurement of the temperature (T2) was performed to assess stress reactivity.

### FST

For the FST mice were carefully put into a water-filled beaker for 6 min to measure the time of immobility (passive floating in the water), swimming and climbing. Time of immobility was used to evaluate depression-like behaviour and swimming/climbing time to measure active stress coping strategies.

### BM

The BM was used to assess learning and memory, following a slightly modified version of the protocol provided by Attar *et al*.^62^. After habituation to the maze, mice were trained to find the target hole (5 training trials), which leads into a hidden escape box. To assess learning capabilities we measured the latency to find the target hole and the number of errors made before identifying the target hole (primary errors). The latency to approach the first hole after starting the test was used to identify any influence of anxiety-like behaviour. Fortyeight h after the last training trial, the hidden escape box was removed and short-term memory was estimated by the time spent in the target quadrant. Seven days later a second probe trial was performed to evaluate long-term memory.

### Molecular analysis

For peptide, protein, and mRNA measurements brains were microdissected as previously described^63^. Brain areas isolated from the left and right hemisphere were collected in separate tubes. One brain half was used for RNA extraction followed by real-time RT PCR, while the other half was used for protein extraction followed by Western Blot or ELISA.

### Electron microscopy

Tissue preparation for electron microscopy and analysis of ultrastructural data followed our previously published procedure^48^. The numerical densities of synapses and dense-core vesicles (DCV) were assessed using a dissector of 5.5 × 5.5 μm size, while the mean length of the presynaptic membrane cross section and of the postsynaptic density in cross section, average width of the synaptic cleft as well as the average number of docked vesicles (vesicles with a maximum distance from the presynaptic membrane of one vesicle diameter) and undocked vesicles (those with a maximum distance of one vesicle diameter from docked or other undocked vesicles at the same synapse) were determined on single sections (10 synapses/animal). The ObjectJ platform of ImageJ was used to measure these parameters.

### Statistics

Statistical data analysis was performed on SPSS 22 (SPSS Inc., Chicago, IL, USA) and SigmaPlot 13 (Systat Software Inc, San Jose, CA, USA). Group differences were assessed by Student’s t-test (EE cage behaviour as well as NPY mRNA and peptide analysis), repeated-measures ANOVA (BM training) or two-way ANOVA (all other data sets). Post-ANOVA analysis of group differences was performed by Tukey’s post hoc comparison. The homogeneity of variances was assessed with the Levene test and normal distribution of the data was evaluated by the Shapiro-Wilk test. Not normal distributed data and data with unequal variances were log-transformed to meet ANOVA assumptions. Probability values ≤ 0.05 were regarded as statistically significant and p-values ≤ 0.1 were regarded as trends. All data are presented as means ± SEM, n referring to the number of mice in each group.

A detailed description of the materials and methods used is provided in the Supplementary Information.

## Additional Information

**How to cite this article**: Reichmann, F. *et al*. Environmental enrichment induces behavioural disturbances in neuropeptide Y knockout mice. *Sci. Rep.* **6**, 28182; doi: 10.1038/srep28182 (2016).

## Supplementary Material

Supplementary Movie S1

Supplementary Movie S2

Supplementary Information
