# Supplementary material for: Environmental enrichment induces behavioural disturbances in neuropeptide Y knockout mice
Source: Sci Rep. 2016 Jun 16;6:28182. doi: 10.1038/srep28182 (PMC4910086; doi:10.1038/srep28182)
Supplement: Supplementary Information [file srep28182-s3.pdf]

Supplementary information

Environmental enrichment induces behavioural disturbances in neuropeptide Y knockout mice

Florian Reichmann[a], Vanessa Wegerer[b], Piyush Jain[a], Raphaela Mayerhofer[a], Ahmed M Hassan[a], Esther E Fröhlich[a], Elisabeth Bock[b], Elisabeth Pritz[b], Herbert Herzog[c], Peter Holzer[a], Gerd Leitinger[b]*

[a]Research Unit of Translational Neurogastroenterology, Institute of Experimental and Clinical Pharmacology, Medical University of Graz, Universitätsplatz 4, 8010 Graz, Austria

[b]Research Unit Electron Microscopic Techniques, Institute of Cell Biology, Histology and Embryology, Medical University of Graz, Harrachgasse 21, 8010 Graz, Austria

[c]Neuroscience Division, Garvan Institute of Medical Research, 384 Victoria Street, Darlinghurst, Sydney, NSW 2010, Australia

*Correspondence: GL (gerd.leitinger@medunigraz.at)

Tel.: +43 316 380 4237; Fax.: +43 316 380 9625

Supplementary Materials and Methods

EPM. For the EPM mice were placed in the centre of a maze consisting of a central platform (5 x 5 cm), two opposing open arms (30 cm long and 5 cm wide), and two opposing closed arms of the same size but equipped with 15-cm high walls at their sides and the far end[1]. At the beginning of each trial, the animals were placed on the central platform facing an open arm. The behaviour of the animals during a 5-min test period was videotaped and tracked with VideoMot 2 software (TSE Systems, Bad Homburg, Germany). Number of entries into and time spent on the open and closed arms as well as total distance travelled was quantified to evaluate anxiety, exploration and locomotion.

OF. The OF consisted of a box (50 x 50 x 30 cm) made of opaque grey plastic. The ground area of the box was divided into a 36 x 36 cm central area and the surrounding border zone[2]. Mice were individually placed in the centre of the OF, and their behaviour during a 5-min test period was videotaped and tracked with the VideoMot 2 software. Number of entries into and time spent in the central area were used as anxiety readouts, while total distance travelled was measured to analyse locomotion.

Stress-induced hyperthermia. This test consisted of a series of 2 rectal temperature measurements[3]. For this, the basal rectal temperature (T1) was measured with a digital thermometer (BAT-12, Physitemp Instruments, Clifton, New Jersey, USA) equipped with a rectal probe for mice representing a physical stressor. Fifteen min later a second measurement of the temperature (T2) was performed. T2 was regarded as a measure of stress reactivity.

FST. For the FST we used a glass beaker (inner diameter 18 cm, height 27 cm) containing tap water at 25 °C[2]. The water was 20 cm deep, which prevented the mice from touching the bottom of the beaker. Mice were carefully put into the water-filled beaker for 6 min to measure the time of immobility (passive floating in the water), swimming and climbing. For this, each session was

videotaped and manually scored by a trained investigator blind to the treatment. Time of immobility was used to evaluate depression-like behaviour.

BM. The BM consisted of a 0.8 cm thick white PVC circular board (diameter: 91 cm) with 20 equally-spaced holes (diameter: 5 cm) at a distance of 2.5 cm from the edge. This apparatus was positioned 61.5 cm above floor level and a black escape box (11 x 5 x 5 cm), to which the mice gained access by stepping down through the target hole, could be fitted below any of the holes. To evaluate learning and memory a slightly modified version of the protocol provided by Attar et al.[4] was used. Briefly, on day 1 of a 2-week test period mice were habituated to the maze by placing them on the board under a clear glass beaker. Mice under the beaker were then carefully guided to the target hole, which leads into the escape box, and allowed to enter on their own. If the mice did not enter, the way to enter was shown to them by the investigator. To avoid potential odour cues the maze and box were thoroughly cleaned after each trial and the position of the target box was changed as well. On the next 2 days (training phase) mice were allowed to freely explore the maze in 3 (day 2) and 2 (day 3) 2-min trials. To assess learning capabilities we measured the latency to find the target hole and the number of errors before identifying the target hole (primary errors). If the mice did not find the target hole, they were again carefully guided to the target hole under the glass beaker. The latency to approach the first hole was used to identify any influence of anxiety-like behaviour. Four different visual cues were mounted on each wall of the testing room to facilitate learning. Fortyeight h after the last training trial the hidden escape box was removed and short-term memory was estimated by the time spent in the target quadrant (tracked with VideoMot 2 software). Seven days after the first probe trial, a second probe trial was performed to evaluate long-term memory.

Cage behaviour. For this test, mice were put individually into a polycarbonate cage of size IV measuring 59.0 x 38.0 x 20.0 cm (length x width x height) and video recorded for 10min. The cage contained 5 items: a running wheel (Dehner, Graz, Austria), a wooden tunnel (Dehner)[5], a hay tunnel (Dehner) and two transparent red plastic mouse houses (Ehret, Tulln, Austria). The time each object was used by the mice was quantified.

CORT ELISA. Circulating CORT levels were determined with an enzyme immunoassay kit (Assay Designs, Ann Arbor, Michigan, USA). For this, mice were euthanized with an overdose of pentobarbital (150 mg/kg IP) and blood samples were collected by cardiac puncture with syringes containing sodium citrate (3.8 %; w/v) as an anticoagulant. Following centrifugation for 10 min at 4 °C and 7000 rpm, blood plasma was stored at -20 °C until assay. The assay was performed according to the manufacturer's instructions. According to the manufacturer's specifications, the sensitivity of the assay is 27 pg/ml, and the intra- and inter-assay coefficient of variation amounts to 7.7 and 9.7 %, respectively.

Mouse brain microdissection. For peptide, protein and mRNA measurements brains were microdissected as previously described[6]. Briefly, fresh frozen mouse brains were cut manually into approximately 1 mm thick coronal slices from which brain areas of interest were isolated on a cold plate (Weinkauf Medizintechnik, Forchheim, Germany) set at -20 °C under a stereomicroscope. Hippocampal tissue was collected across the whole rostrocaudal extent starting from the limit of the hippocampal formation (Bregma: -0.94) to the caudal end of the dentate gyrus (Bregma: -4.04). The microdissection of the amygdala was also performed across the whole rostrocaudal extent of the amygdalar complex (Bregma: -0.58 to Bregma: -2.54) and thus the tissue taken contained all major amygdalar subnuclei (medial, central, basolateral, basomedial, lateral and cortical). Isolated brain areas were collected in homogenization tubes kept on dry ice and stored at -70 °C until further processing. Areas isolated from the left and right hemisphere were collected in separate tubes. One brain half was used for RNA extraction, while the other half was used for protein extraction. To exclude lateralization effects, a random number generator was used to allocate the right or the left brain half to RNA or protein extraction, respectively.

Protein extraction. Brain areas used for protein and peptide measurements were homogenized in lysis buffer (50 mM Tris-HCl pH 8, 150 mM NaCl, 1 % (v/v) Triton X-100, 0.5 % (v/v) sodium deoxycholate and 10 mM PMSF) using a Peqlab Precellys 24 homogenizer (Peqlab, Erlangen, Germany) [7]. The tissue homogenates were centrifuged (10,000 rpm, 4 °C, 10 min) to pellet debris and

the protein content was measured with the BCA protein assay kit (Pierce Biotechnology, Rockford, IL, USA).

Western Blot. For Western blot, protein lysates (10 or 50 µg; amygdala or hippocampus) were mixed with 4x NuPAGE LDS sample buffer and NuPAGE sample reducing agent, heated for 10 min at 70 °C and loaded on 4-12 % gradient SDS-PAGE gels (Life Technologies, Carlsbad, CA, USA) for electrophoresis[7]. After transfer to nitrocellulose membranes (45 µm; Life Technologies), the membranes were blocked with non-fat milk (5 % w/v) in Tris-buffered saline containing Tween20 (TBS) for 1 h. After being washed, membranes were incubated overnight at 4°C with anti-NR3C1 antibody (1:1000, Cell Signalling, D6H2L) diluted in TBS containing bovine serum albumin (5 % w/v). After another wash, the membranes were incubated with horseradish peroxidase-conjugated goat anti rabbit IgG (1:200,000, Biomol). Immunoreactive bands were visualized with Clarity™ Western ECL Blotting Substrate (Biorad, Hercules, CA, USA) and detected by the ChemiDoc™ Touch Imaging System (Bio-Rad). For normalization, membranes were incubated with anti-glyceraldehyde 3-phosphate dehydrogenase (GAPDH) antibody (1:1000, Santa Cruz, SC-25778). Densitometric evaluations of immunoreactive bands were performed with the Image Lab software volume module (Biorad).

BDNF ELISA. Amygdalar and hippocampal BDNF concentrations were measured using the BDNF E_{max}® ImmunoAssay System (Promega, Madison, WI, USA). The assay was performed according to the manufacturer’s instructions using protein lysates diluted in assay buffer (1:10). The company reports a sensitivity of 15.6 pg/ml and a cross reactivity with other related neurotrophic factors of less than 3 % for this assay.

NPY fluorescence EIA. NPY concentrations were measured by fluorescence EIA (Phoenix Pharmaceuticals, Burlingame, CA, USA). For this, NPY was extracted from the amygdalar and hippocampal protein lysates. Protein (200 or 500 µg) was extracted in 2N acetic acid, respectively, and centrifuged for 10 min at 2400 rpm/ 4 °C[8]. The supernatants were lyophilized and stored at -70

°C. For the assay the lyophilisate was reconstituted in assay buffer and the EIA was performed according to the manufacturer's instructions. The sensitivity of the assay is 11.9 pg/ml and there is no cross reactivity with peptide YY or pancreatic polypeptide.

Real-time RT PCR. Brain halves used for real-time RT PCR were homogenized in QIAzol lysis reagent. RNA was extracted with the RNeasy Lipid Tissue Mini kit (Qiagen, Hilden, Germany) and reverse transcribed using the high capacity cDNA reverse transcription kit (Applied Biosystems, FosterCity, CA, USA)[6]. For relative quantification of mRNA levels within a given brain area, specific primers for *Npy* (forward 5' -> 3': CAGATACTACTCCGCTCTGCGACACTACA; reverse 5' -> 3': TTCCTTCATTAAGAGGTCTGAAATCAGTGTC), *Bdnf* (forward 5' -> 3': GTGACAGTATTAGCGAGTGG; reverse 5' -> 3': TTCTCTAGGACTGTGACCGT), *Nr3c1* (forward 5' -> 3': GACTCCAAAGAATCCTTAGCTCC; reverse 5' -> 3': CTCCACCCCTCAGGGTTTTAT), *Gapdh* (Qiagen, Mm_Gapdh_3_SG QuantiTectPrimerAssay), phosphoglycerate kinase 1 (*Pgk1*; forward 5' -> 3': ATGTCGCTTTCCAACAAGCTG; reverse 5' -> 3': GCTCCATTGTCCAAGCAGAAT) and peptidylprolyl isomerase A (*Ppia*; forward 5' -> 3': TTCCAGGATTCATGTGCCAG; reverse 5' -> 3': CCATCCAGCCATTCAGTCTT) were used at a concentration of 1 µM each together with the SsoAdvanced Universal SYBR Green Supermix (Bio-Rad). DNA sequencing and BLAST analysis confirmed the amplification of the correct base pair sequence. Samples were measured in triplicates on a LightCycler 480 System (Roche, Rotkreuz, Switzerland). Quantitative measurement of target gene levels relative to controls was performed with the $2^{-\Delta\Delta Ct}$ method. *Gapdh*, *Pgk1* and *Ppia* were used as reference genes.

Electron microscopy. Tissue preparation for electron microscopy followed our previously published procedure[9]. In brief, excised mouse brains were cut in half at the midline, and immersion-fixed for 48 h in 2 % formaldehyde, 2.5 % glutardialdehyde in 0.1 M cacodylate buffer pH 7.4 at 4 °C and rinsed in the same buffer for at least 24 h. Coronal vibratome sections were made on a Leica VT 1000 vibratome until the DGpl was reached, verified by comparing with thionine-stained vibratome sections and the mouse brain atlas of Franklin and Paxinos[10]. A vibratome section close to Bregma -

1.3 was cut at 150 µm thickness, post fixed in 2 % osmium tetaraoxide solution, dehydrated in a series of graded alcohols, and embedded in TAAB embedding resin (TAAB, Berkshire, UK). Three coherent semi thin sections were made at a thickness of 500 nm and stained with 1 % toluidine blue solution, of which the first and third were used for counting the number of cells in the DGpl. Immediately adjacent to these semi thin sections, serial thin sections were made at 55 nm thickness, of which one pair was used for further analysis. These thin sections were examined with a Tecnai G2 20 electron microscope (FEI, Eindhoven, Netherlands) operating at 120 kV with a US 1000 digital camera (Gatan, Pleasanton, USA). For analysis, a minimum of 20 image pairs were made from two adjacent sections in each animal, using several software packages as previously described[9]. Serial EM software (version 3.3.1) was used to obtain and merge 4 adjacent camera images at 5000x magnification. FEI Serial Section Software (FEI, Eindhoven Netherlands) was used to store the location of each point on the first image of each pair and to retrieve its location on the second image, where another montage was obtained using Serial EM. The numerical densities of synapses and DCV were assessed using a dissector of 5.5 x 5.5 µm size. Furthermore, on 10 synapses/animal, the following synaptic features were determined on single sections: mean length of the presynaptic membrane cross section and of the postsynaptic density in cross section, average width of the synaptic cleft as well as the average number of docked vesicles (vesicles with a maximum distance from the presynaptic membrane of one vesicle diameter) and undocked vesicles (those with a maximum distance of one vesicle diameter from docked or other undocked vesicles at the same synapse) within the thin section. The ObjectJ platform of ImageJ was used to measure these parameters. All measurements and counts were made on coded samples by an experimenter blind to the treatment groups.

Supplementary Figure

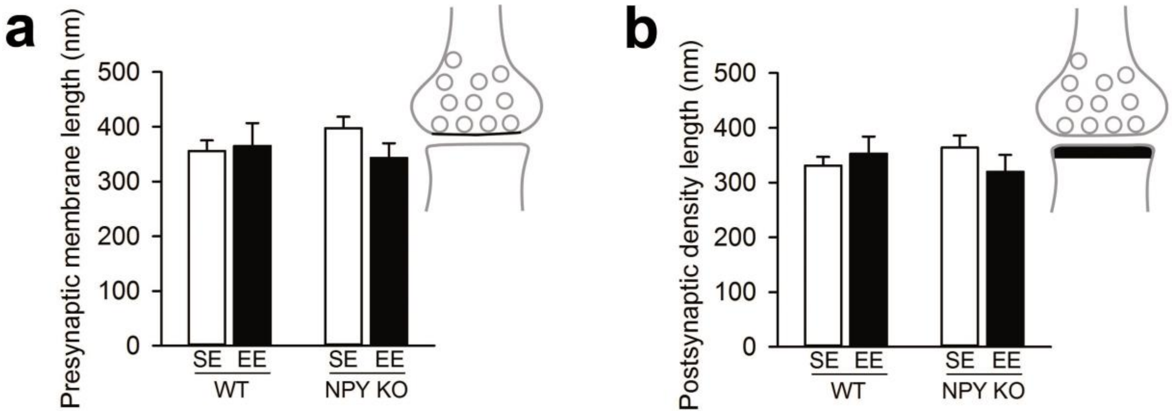

Fig. S1. EE and NPY KO do not alter (A) presynaptic membrane length and (B) postsynaptic density length of synapses in the DGpl. *Values were measured in WT and NPY KO mice kept in EE or SE and represent mean ± SEM. Data in A and B are derived from 10 synapses/animal and 4-5 animals/group.*

Supplementary Movies

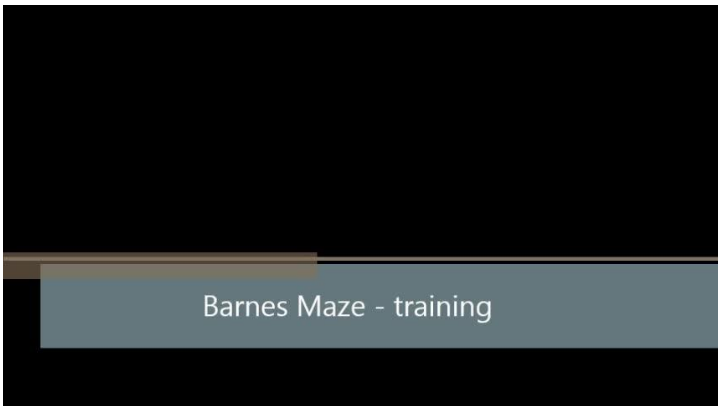

Movie S1. Representative behaviour of WT and NPY KO mice during the first training trial of the Barnes Maze test. *NPY KO mice move very slowly and tend to freeze in the centre of the maze. In contrast, WT mice immediately begin to explore the maze after starting the training trial.*

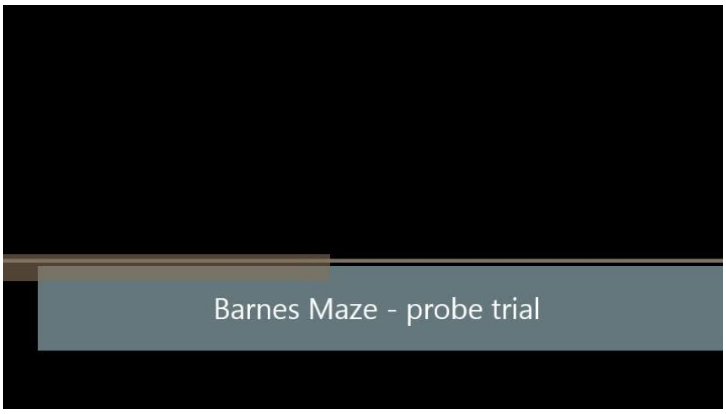

Movie S2. Representative behaviour of EE-housed and SE-housed NPY KO mice during the probe trial of the Barnes maze test. *EE-housed NPY KO mice do not remember the location of the target hole and thus employ a random search strategy. SE-housed NPY KO mice remember the location of the target hole and stay in the target quadrant.*

References

1. Brunner, S. M. *et al*. GAL3 receptor KO mice exhibit an anxiety-like phenotype. *Proc. Natl. Acad. Sci. U. S. A.* **111**, 7138-7143 (2014).

2. Farzi, A. *et al*. Synergistic effects of NOD1 or NOD2 and TLR4 activation on mouse sickness behavior in relation to immune and brain activity markers. *Brain Behav. Immun.* **44**, 106-120 (2015).

3. Olivier, B. *et al*. Stress-induced hyperthermia and anxiety: pharmacological validation. *Eur. J. Pharmacol.* **463**, 117-132 (2003).

4. Attar, A. *et al*. A shortened Barnes maze protocol reveals memory deficits at 4-months of age in the triple-transgenic mouse model of Alzheimer's disease. *PLoS One* **8**, e80355 (2013).

5. Reichmann, F., Painsipp, E. & Holzer, P. Environmental Enrichment and Gut Inflammation Modify Stress-Induced c-Fos Expression in the Mouse Corticolimbic System. *PLoS One* **8**, e54811 (2013).

6. Reichmann, F. *et al*. Dextran sulfate sodium-induced colitis alters stress-associated behaviour and neuropeptide gene expression in the amygdala-hippocampus network of mice. *Sci. Rep.* **5**, 9970 (2015).

7. Jain, P. *et al*. Behavioral and molecular processing of visceral pain in the brain of mice: impact of colitis and psychological stress. *Front. Behav. Neurosci.* **9**, 177 (2015).

8. Amann, R., Sirinathsinghji, D. J., Donnerer, J., Liebmann, I. & Schuligoi, R. Stimulation by nerve growth factor of neuropeptide synthesis in the adult rat in vivo: bilateral response to unilateral intraplantar injections. *Neurosci. Lett.* **203**, 171-174 (1996).

9. Reichmann, F. *et al*. A novel unbiased counting method for the quantification of synapses in the mouse brain. *J. Neurosci. Methods* **240**, 13-21 (2015).

10. Franklin, K. B. J. & Paxinos, G. in *The Mouse Brain in Stereotaxic Coordinates* (Elsevier/Academic Press, 2008).
